# Botulinum toxin use in patients with neurological disorders: A U.S.‐based claims database analysis

**DOI:** 10.1002/pmrj.70105

**Published:** 2026-03-11

**Authors:** David M. Simpson, Jonathan Bouchard, Simon Page, Seth Goldfarb, Atul T. Patel

**Affiliations:** ^1^ Department of Neurology Icahn School of Medicine at Mount Sinai New York New York USA; ^2^ Ipsen Cambridge Massachusetts USA; ^3^ Ipsen London UK; ^4^ Kansas City Bone & Joint Clinic Overland Park Kansas USA

## Abstract

**Background and Objective:**

Real‐world evidence describing long‐term persistency with botulinum toxin (BoNT) therapy is limited. We assessed treatment patterns and persistency with BoNT in clinical practice over 2 years.

**Design:**

Retrospective, longitudinal, claims database analysis.

**Setting:**

Medical claims data from the Merative MarketScan database in the United States.

**Participants and Intervention:**

Patients with an index medical claim for any BoNT therapy between October 1, 2016 and October 31, 2019, and at least 2 follow‐up years.

**Main Outcomes Measures:**

Number of treatments, time between treatments, and persistency (based on a 120‐day look‐forward from treatment date) were evaluated over the 2‐year follow‐up period. Using the 120‐day look‐forward from treatment date, 31.1% of all patients (*n* = 19,711) received only one BoNT injection cycle in the 2‐year follow‐up, indicating a notable drop in the proportion of patients who persisted with BoNT treatment. This prompted further analysis of cohorts by indication: patients with adult spasticity (*n* = 1912), pediatric spasticity (*n* = 638), stroke (*n* = 611), cervical dystonia (*n* = 688), migraine (*n* = 7778), or bladder dysfunction (*n* = 1791) diagnoses. [Correction added on 02 April 2026, after first online publication: In the previous sentence, “decrease” has been changed to “drop” in this version.]

**Results:**

A large proportion of patients treated with BoNT stopped after 1–2 cycles. Across cohorts, the mean (SD) number of treatments administered was 2.1–4.7 (1.0–3.0). The time between treatments (80th percentile reported) ranged from 105 days to 319 days depending on the cohort.

**Conclusions:**

Increased understanding of BoNT treatment persistency in clinical practice is essential to help to optimize patient care. This study opens the discussion and field for future studies about why many patients discontinue BoNT therapy after only one or two treatment cycles. Further research will help to determine the underlying reasons for cessation of treatment.

## INTRODUCTION

Botulinum toxin A (BoNT‐A) is widely used in the long‐term management of neurological disorders, including adult and pediatric spasticity, cervical dystonia, chronic migraine, and bladder dysfunction.^1^, ^2^, ^3^ BoNT‐B is indicated in the United States for the treatment of cervical dystonia in adults in addition to chronic sialorrhea, a frequent symptom of neurological diseases.^4^ The conditions treated with BoNT therapy are characterized by increased muscle tone and abnormal movement and/or pain that may cause physical and emotional disability and affect patient quality of life.^5^, ^6^, ^7^ BoNT acts by blocking acetylcholine release at the neuromuscular junction, thereby reducing muscle hyperactivity,^8^ and, in the case of chronic migraine, BoNT is thought to interfere with pain signaling.^9^ Owing to the reversible pharmacological effect of BoNTs, repeated treatment cycles are necessary to maintain satisfactory therapeutic outcomes,^8^ and must follow indication‐specific regimens as described in this article.

BoNT‐A has an established safety profile^10^, ^11^ and is recommended as a first‐line treatment for spasticity by several national and international guidelines.^8^, ^12^, ^13^ Spasticity etiologies include cerebral palsy, multiple sclerosis, traumatic brain injury, and stroke; up to 40% of patients experience paresis after a stroke.^8^, ^14^, ^15^, ^16^ Importantly, one in four people aged over 25 years will have a stroke in their lifetime,^17^ and a meta‐analysis of epidemiological studies showed that poststroke spasticity (PSS) occurred in 25% of patients after a stroke and 40% of patients with paresis after a stroke.^15^ Several guidelines also recommend BoNT‐A and BoNT‐B therapy as first‐line treatment for adult patients with cervical dystonia.^13^, ^18^, ^19^ OnabotulinumtoxinA (onaBoNT‐A) is recommended for the prophylaxis of chronic migraine.^1^ In bladder dysfunction, BoNT‐A is recommended for patients who have an inadequate response to or are intolerant of an anticholinergic medication.^1^ In all indications, BoNT retreatment should occur based on the return of clinical symptoms^1^, ^2^ and must not be given more frequently than every 12 weeks, or every 16 weeks for pediatric upper limb spasticity.^1^, ^2^, ^3^, ^4^

Adherence to prescribed treatment is critical to successfully meet the needs and goals of patients,^20^ and retreatment should be tailored to individual patients based on treatment response and return of clinical symptoms.^1^, ^2^, ^3^, ^4^ Despite the important role that BoNT therapy can play in the management of various physically and psychologically debilitating neurological conditions,^8^, ^13^, ^18^ studies evaluating the real‐world duration of BoNT therapy and long‐term adherence to treatment are limited.^21^ This retrospective, longitudinal study, with a 3‐year index period and a 2‐year follow‐up period, described BoNT treatment patterns and persistency in real‐world clinical practice in a commercially insured population in the United States.

## METHODS

### Study design and data source

This retrospective, longitudinal study explored persistency with BoNT treatment over a 2‐year follow‐up period. It used U.S. medical claims data from the Merative MarketScan database (formerly the IBM MarketScan database) among patients with a first medical claim for BoNT between October 1, 2016 and October 31, 2019 (the index period) (Figure 1). The index date was defined as the date of the first BoNT administration. All commercially available preparations of BoNT serotypes A and B in the United States were included: onaBoNT‐A, abobotulinumtoxinA (aboBoNT‐A), incobotulinumtoxinA, and rimabotulinumtoxinB. A 12‐month washout period before the index date was used to identify incident BoNT use and only patients with a BoNT medical claim in that period were included.

**FIGURE 1:**
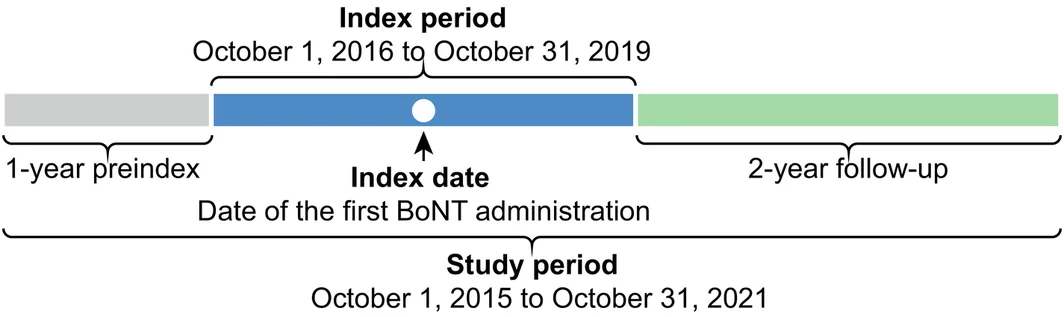
Study design schematic. BoNT, botulinum toxin.

The Merative MarketScan database is a large U.S. commercial claims database with information on approximately 293 million patients. It comprises detailed cost, resource use, and patient data for health care services performed in both inpatient and outpatient settings for patients insured commercially or as part of the national Medicare program. Medical claims are linked to outpatient prescription drug claims and person‐level enrollment data. The database provides robust, longitudinal data broadly representative of the population of the United States.^22^, ^23^ Diagnoses are coded according to the *International Statistical Classification of Diseases and Related Health Problems Tenth Revision* (ICD‐10). BoNT treatments are coded using the Healthcare Common Procedure Coding System and medical procedures according to the Current Procedural Terminology.

### Study cohorts

Given that this was a retrospective claims study in which all data were deidentified according to regulations, ethics approval and consent to participate were not required. Eligible patients were continuously enrolled in the database for at least 36 months starting during the study period, had a medical claim for BoNT (not product specific) in the index period, and had at least 2 years of subsequent follow‐up data. Adult and pediatric patients who received at least one intramuscular BoNT‐A injection in the index period were identified using the following the Healthcare Common Procedure Coding System codes: J0585, J0586, J0587, and J0588. Patients with any claim for BoNT in the year preceding their index date were excluded to avoid inclusion of incident BoNT use.

For each patient, the diagnosis for which BoNT was indicated was inferred by ICD‐10 codes recorded on the index date or within the index period; patients were stratified into six mutually exclusive cohorts (Figure 2). On‐label indications were those approved in the United States for aboBoNT‐A (see Table 1 for definitions).

**FIGURE 2:**
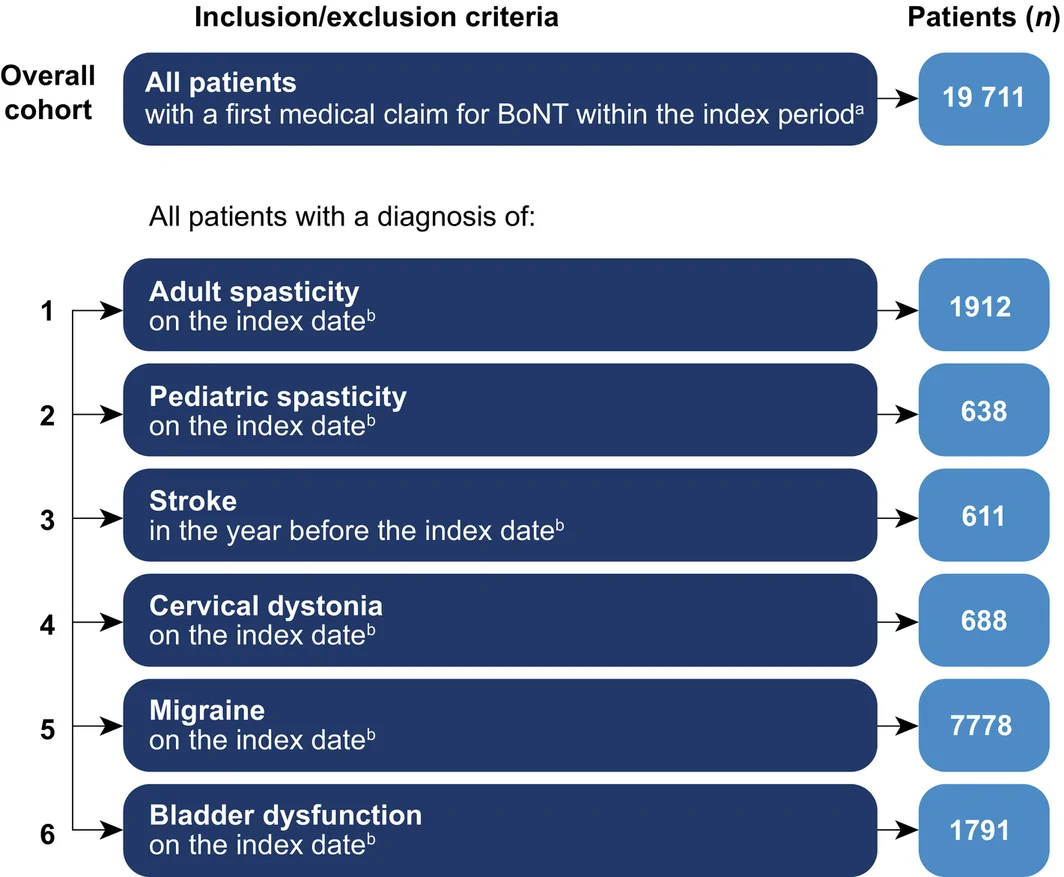
Attrition of the study sample. ^a^Excluding incident patients (no BoNT claim in the 12 months before index date); including patients with ≥36 months of continuous enrollment starting during the study period; ^b^Excluding patients with aboBoNT‐A off‐label^2^ diagnoses on the index date (spasticity, stroke, migraine), in the previous year (cervical dystonia), after the index date (migraine), or any time during the study (bladder dysfunction). aboBoNT‐A, abobotulinumtoxinA; BoNT, botulinum toxin.

**TABLE 1:** On‐label BoNT indications.

Cohort	Study definition
Overall cohort	All patients with a first medical claim for BoNT treatment within the index period (all commercially available preparations of BoNT‐A and BoNT‐B in the United States)
Cohort 1 – Adult spasticity	Adult patients (aged ≥18 years) with an on‐label aboBoNT‐A spasticity diagnosis on the index date and excluded aboBoNT‐A off‐label diagnoses
Cohort 2 – Pediatric spasticity	Pediatric patients (aged <18 years) with an on‐label aboBoNT‐A spasticity diagnosis on the index date and excluded aboBoNT‐A off‐label diagnoses
Cohort 3 – Stroke	Patients with a diagnosis of stroke during the 12 months before the index date and excluded aboBoNT‐A off‐label diagnoses on the index date
Cohort 4 – Cervical dystonia	Patients with a diagnosis of cervical dystonia on the index date and excluded aboBoNT‐A off‐label diagnoses in the previous year
Cohort 5 – Migraine	Patients with a diagnosis of migraine on the index date and excluded aboBoNT‐A off‐label nonmigraine diagnoses on or after the index date
Cohort 6 – Bladder dysfunction	Patients with a diagnosis of bladder dysfunction on the index date and excluded aboBoNT‐A off‐label nonbladder dysfunction diagnoses at any time during the study

Abbreviations: aboBoNT‐A, abobotulinumtoxinA; BoNT‐A, botulinum toxin‐A; BoNT‐B, botulinum toxin‐B.

### Endpoints – measures

The demographic and clinical characteristics were assessed at baseline.^24^ Mean number of treatments, time between treatments, and persistency with BoNT treatment were evaluated for each cohort in the 2‐year follow‐up period. Patients were considered persistent with treatment if they remained on treatment and did not experience a treatment break between two treatment cycles.^25^

For all patients (overall cohort), persistency was assessed using a model in which patients were expected to receive BoNT treatment with a frequency of approximately every 4 months. Persistency was assessed using a 120‐day look‐forward from treatment date, corresponding to a 12‐week period (minimum time recommended between BoNT treatments^1^, ^2^, ^3^, ^4^) with a grace period of approximately 5 weeks. Where persistency in the overall cohort did not fit with the expected model, further analyses would be performed to assess persistency by indication.

Statistical analyses were descriptive and undertaken in each cohort separately. Frequency (*n*) and proportion (%) were reported for categorical variables. Mean, SD, median, minimum and maximum, and 80th percentile were reported for continuous variables. Data analyses were performed using the SAS 9.1 software.

## RESULTS

Baseline demographics are shown in Table 2. In total, 19,711 patients (overall cohort) were included in the analysis. There was a notable drop in the proportion of patients receiving BoNT treatment after the first injection cycle in the overall cohort (Figure 3A, Table 3), with 31.1% of patients who received only one BoNT treatment cycle. This prompted the analysis of the cohorts by indication, to determine which indication(s) may be driving this decrease.

**TABLE 2:** Baseline demographics.

Cohort	Overall (all patients) (*n* = 19,711)	Adult spasticity (*n* = 1912)	Pediatric spasticity (*n* = 638)	Stroke (*n* = 611)	Cervical dystonia (*n* = 688)	Migraine (*n* = 7778)	Bladder dysfunction (*n* = 1791)
Age, y							
Mean (SD)	46.5 (16.9)	50.7 (16.2)	9.3 (4.7)	48.8 (22.6)	54.6 (13.8)	44.7 (12.4)	59.8 (16.6)
Median (IQR)	48.0 (21.0)	53.0 (21.0)	9.0 (8.0)	55.0 (29.0)	56.0 (15.0)	46.0 (17.0)	60.0 (20.0)
Gender, female, *n* (%)	15,129 (76.8)	1165 (60.9)	290 (45.5)	337 (55.2)	484 (70.3)	6680 (85.9)	1462 (81.6)
Payer type, *n* (%)							
Commercial	17,596 (89.3)	1609 (84.2)	638 (100.0)	473 (77.4)	564 (82.0)	7531 (96.8)	1129 (63.0)
Medicare	2115 (10.7)	303 (15.8)	0	138 (22.6)	124 (18.0)	247 (3.2)	662 (37.0)
CCI at baseline, mean (SD)	0.9 (1.5)	1.8 (2.0)	1.9 (1.2)	3.7 (1.9)	0.8 (1.5)	0.6 (1.1)	1.5 (1.8)

Abbreviations: CCI, Charlson Comorbidity Index; IQR, interquartile range.

**FIGURE 3:**
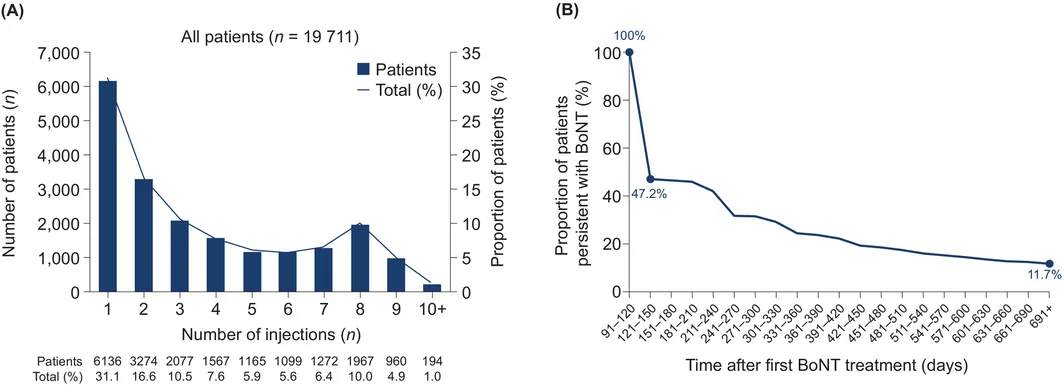
Number of BoNT treatments (A) and persistency (B) in the overall cohort (all patients) based on the 120‐day look‐forward from treatment date in the 2‐year follow‐up period. BoNT, botulinum toxin.

**TABLE 3:** Number of treatments, time between treatments, and persistency with treatment.

Cohort	Overall (all patients) (*n* = 19,711)	Adult spasticity (*n* = 1912)	Pediatric spasticity (*n* = 638)	Stroke (*n* = 611)	Cervical dystonia (*n* = 688)	Migraine (*n* = 7778)	Bladder dysfunction (*n* = 1791)
Number of BoNT treatments over the 2‐year follow‐up period							
Mean (SD)	3.7 (3.0)	3.6 (2.6)	2.6 (1.9)	3.6 (3.0)	3.9 (2.7)	4.7 (3.0)	2.1 (1.0)
Median (IQR)	3.0 (5.0)	3.0 (5.0)	2.0 (3.0)	3.0 (5.0)	3.0 (6.0)	4.0 (6.0)	2.0 (2.0)
Time between BoNT treatments							
80th percentile reported, days (min, max)	126 (1, 729)	126 (1, 722)	217 (1, 723)	129 (1, 677)	123 (1, 725)	105 (1, 727)	319 (1, 726)
Proportion of patients who received BoNT treatment, *n* (%)							
Only 1	6136 (31.1)	588 (30.8)	273 (42.8)	165 (27.0)	197 (28.6)	1465 (18.8)	804 (44.9)
1–2	9410 (47.7)	940 (49.2)	398 (62.4)	276 (45.2)	308 (44.8)	2589 (33.3)	1251 (69.8)
≥3	10,301 (52.3)	948 (49.6)	240 (37.6)	335 (54.8)	376 (54.7)	5189 (66.7)	540 (30.2)
8–9	2927 (14.9)	199 (10.4)	15 (2.4)	56 (9.2)	106 (15.4)	1923 (24.7)	6 (0.3)
Proportion of patients persistent with BoNT treatment, based on the 120‐day look‐forward from treatment date							
Remained on treatment at 120 days, *n* (%)	9298 (47.2)	938 (49.1)	115 (18.0)	281 (46.0)	352 (51.2)	5353 (68.8)	115 (6.4)
Remained on treatment at 690 days, *n* (%)	2310 (11.7)	156 (8.2)	14 (2.2)	44 (7.2)	85 (12.4)	1534 (19.7)	6 (0.3)

Abbreviations: BoNT, botulinum toxin; IQR, interquartile range; max, maximum; min, minimum.

Patients within therapeutic groups of interest from the overall cohort were selected for further analysis, based upon ICD‐10 diagnostic code criteria (Table 1). The resulting cohorts were adult spasticity (*n* = 1912), pediatric spasticity (*n* = 638), stroke (*n* = 611), cervical dystonia (*n* = 688), migraine (*n* = 7778), and bladder dysfunction (*n* = 1791). The mean (SD) age at the start of treatment in the overall cohort was 46.5 (16.9) years and 76.8% of patients were female. The mean (SD) age for the adult spasticity cohort was 50.7 (16.2) years and 9.3 (4.7) years for the pediatric spasticity cohort. The mean age (SD) in the other cohorts was 59.8 (16.6) years for bladder dysfunction, 54.6 (13.8) years for cervical dystonia, 48.8 (22.6) years for stroke, and 44.7 (12.4) years for migraine. The majority of patients in the migraine (85.9%), bladder dysfunction (81.6%), cervical dystonia (70.3%), adult spasticity (60.9%), and stroke (55.2%) cohorts were female. Fewer than half (45.5%) of patients in the pediatric spasticity cohort were female. The mean (SD) Charlson Comorbidity Index^26^ was notably higher in the stroke cohort (3.7 [1.9]) than the other cohorts (range of 0.6 [1.1]–1.9 [2.0]), indicating a higher risk of death from comorbid disease in patients with stroke (Table 2).

The decrease in the proportion of patients receiving BoNT treatment after the first injection cycle was observed across all cohorts evaluated (Figure 4). The proportion of patients receiving only one cycle was highest for the bladder dysfunction (44.9%) and pediatric spasticity (42.8%) cohorts, followed by the adult spasticity (30.8%), cervical dystonia (28.6%), stroke (27.0%), and migraine (18.8%) cohorts.

**FIGURE 4:**
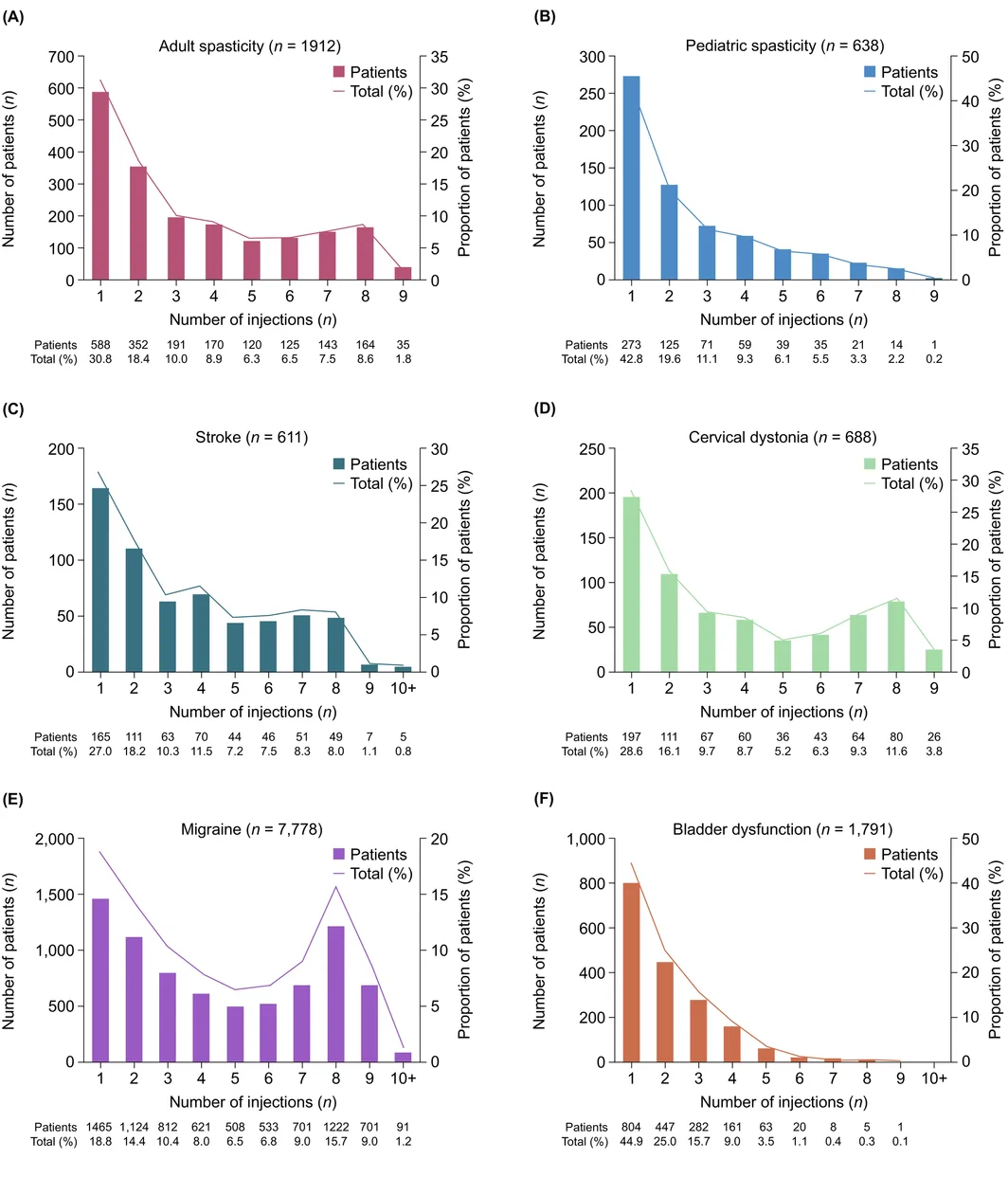
Number of BoNT treatments per cohort in the 2‐year follow‐up period. A, adult spasticity; B, pediatric spasticity; C, stroke; D, cervical dystonia; E, migraine; F, bladder dysfunction. BoNT, botulinum toxin.

In the overall cohort there was a peak in the proportion of patients receiving eight to nine BoNT treatment cycles (14.9%) over the 2‐year follow‐up period (Figure 3A). In the indication cohorts, the proportion of patients who received eight to nine treatments over 2 years (ie, once every 3–4 months) was 10.4% for adult spasticity, 15.4% for cervical dystonia, 9.2% for stroke, and 24.7% for migraine (Figure 4, Table 3).

The number of treatments and time between treatments are shown in Table 3. In the overall cohort, the mean (SD) number of BoNT treatments over the 2‐year follow‐up period was 3.7 (3.0), and the time between treatments (80th percentile reported) was 126 days. In the indication cohorts, the mean (SD) number of BoNT treatments over the 2‐year follow‐up period ranged from 2.1 (1.0) for bladder dysfunction to 4.7 (3.0) for migraine. The time between treatments (80th percentile reported) ranged from 105 to 319 days; it was broadly similar for the migraine, cervical dystonia, adult spasticity, and stroke cohorts (105, 123, 126, and 129 days, respectively), and longer for the bladder dysfunction and pediatric spasticity cohorts (319 and 217 days, respectively).

Using the 120‐day look‐forward from treatment date, 47.2% of patients in the overall cohort were considered persistent with BoNT after the first treatment cycle, with similar findings in the adult spasticity and stroke cohorts (Figures 4 and 5, Table 3). The proportion of patients was lowest in the bladder dysfunction (6.4%) and pediatric spasticity (18.0%) cohorts, and highest in the migraine cohort (68.8%).

**FIGURE 5:**
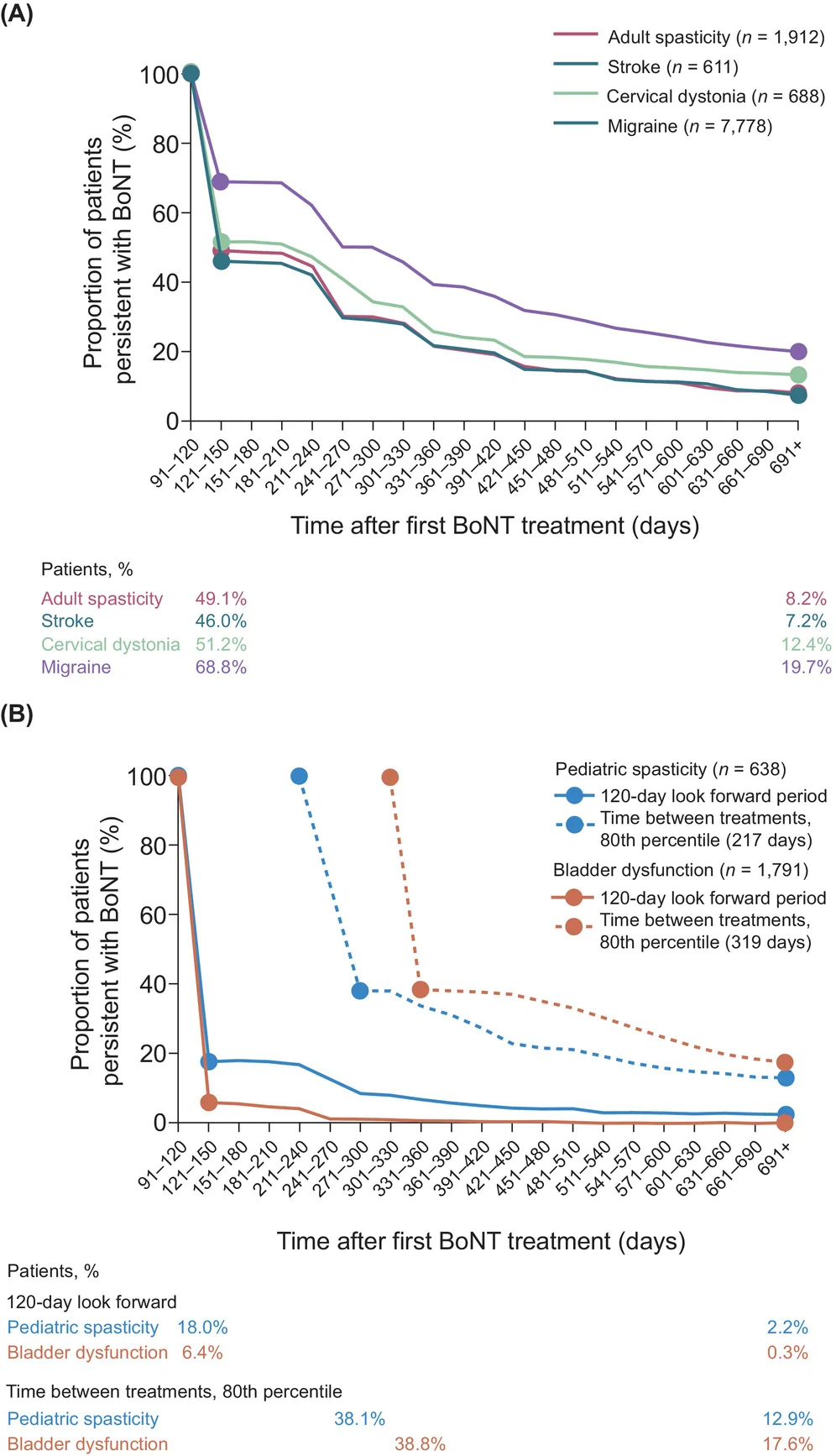
Persistency curves per cohort in the 2‐year follow‐up period. Persistency was assessed in all cohorts based on a 120‐day look‐forward from treatment date (A) and on the time between treatment determined in this study (80th percentile value of days to next treatment) for the pediatric spasticity and bladder dysfunction cohorts (B). Note, the observations for each cohort were independent and unadjusted (not directly comparable). BoNT, botulinum toxin.

Over the 2‐year follow‐up period, the proportion of patients considered persistent with BoNT was only 11.7% in the overall cohort and <10% in the adult spasticity and stroke cohorts. The proportion of patients considered to be persistent was lowest in the bladder dysfunction (0.3%) and pediatric spasticity (2.2%) cohorts, and highest in the migraine cohort (19.7%).

Given the low persistency with BoNT treatment observed in the bladder dysfunction and pediatric cohorts using the 120‐day look‐forward period, the time between treatments (80th percentile reported) was also used to assess persistency. The proportion of patients considered persistent with treatment after the first treatment cycle was 38.8% in the bladder dysfunction and 38.1% in the pediatric spasticity cohorts. Over the 2‐year follow‐up period, the proportion of patients considered persistent with treatment was 17.6% in the bladder dysfunction and 12.9% in the pediatric spasticity cohorts.

A comparison of persistency by BoNT‐A product was not possible owing to many patients having potentially received treatment with more than one BoNT‐A product; sample sizes for the individual products were too low to permit meaningful comparison.

## DISCUSSION

This retrospective, longitudinal study used a nationally representative health care claims database from the United States to characterize BoNT treatment persistency over a 2‐year follow‐up period using a 120‐day look‐forward from treatment date. Persistency with BoNT treatment was low across all cohorts and there was a notable drop in the proportion of patients receiving BoNT treatment after the first injection cycle. [Correction added on 02 April 2026, after first online publication: In the previous sentence, “decrease” has been changed to “drop” in this version.]

The gender and mean age of patients were in line with expectations for each cohort. Most patients in the migraine cohort were female (85.9%) and the mean age was 44.7 years. Multiple epidemiological studies have found that women are up to three times more likely to be affected by migraine than men,^27^, ^28^ and the prevalence peaks among women in midlife.^29^ Studies have also shown that urinary incontinence is three times more common in women than in men,^30^ often in the perimenopausal period.^31^ Additionally, the majority of patients with overactive bladder syndrome are female, and the mean age is 60.1 years.^32^ In terms of movement disorders, females in this study represented 70.3%, 60.9%, and 45.5% of patients in the cervical dystonia, adult spasticity, and pediatric spasticity cohorts, respectively, and the mean ages were 54.6, 50.7, and 9.3 years, respectively. This was consistent with the findings of another real‐world study from the United States in which most patients in the cervical dystonia (69.5%) and adult spasticity (54.7%) cohorts were female; fewer than half of patients in the pediatric spasticity (42.4%) cohort were female. The mean age was 52.9 years for the cervical dystonia cohort, 46.9 years for the adult spasticity cohort, and 8.9 years for the pediatric spasticity cohort.^33^ The mean age in the stroke cohort was younger than expected (48.8 years), possibly reflecting the age profile of the majority of patients who were commercially insured (versus those with Medicare coverage).^34^

Using a 120‐day look‐forward from treatment date, over the 2‐year follow‐up period, 30.8% of adult patients with spasticity, 27.0% of patients with stroke, and 28.6% of patients with cervical dystonia received only one BoNT injection. Analysis of data from the French Programme de Médicalisation des Systèmes d'Information database found that among 138,481 patients with spasticity treated with BoNT‐A between 2014 and 2020, 36.6% received only one BoNT‐A injection. Additionally, only 2.3% of survivors of stroke with PSS (*n* = 360,740) received more than one BoNT‐A treatment cycle, with fewer than 1% receiving BoNT‐A injections once every 3–4 months.^35^ Of potential interest, a German study on long‐term adherence to BoNT‐A therapy (*n* = 1351) reported significantly shorter treatment adherence among patients with spasticity than among patients with cervical dystonia.^7^

Adult patients with spasticity or cervical dystonia usually require repeated BoNT‐A intramuscular injections once every 12–16 weeks.^1^, ^2^, ^3^, ^4^ Based on this expected retreatment regimen, the mean number of treatments over the 2‐year period was lower and the time between treatments was longer than anticipated for the cervical dystonia, adult spasticity, and stroke cohorts. Our results for these cohorts are in line with previous findings in age‐ and gender‐matching populations reporting means of 115.5 and 124.1 days between treatments for their cervical dystonia and adult spasticity cohorts, respectively.^33^ Tailoring BoNT treatment regimens to individual patient needs, response, and clinical symptom recurrence is recommended,^1^, ^2^, ^3^, ^4^ and good communication between patients and an experienced multidisciplinary team is required to optimize management.^36^ As such, spasticity treatment often combines pharmacological and physical management to accommodate patient needs.^37^ When physical therapy helped patients to achieve treatment goals, they may not have required BoNT retreatment in the 12–16 weeks following their first treatment.

In the pediatric spasticity cohort, 12.9% of patients were considered persistent with treatment over the 2‐year period using the time between treatment determined in this study (217 days), which may represent a typical retreatment schedule in these patients. The time to retreatment tends to be longer than once every 3–4 months in pediatric patients with lower limb spasticity (every 16–22 weeks) and with upper limb spasticity (every 12–28 weeks) than in adult patients.^1^, ^2^, ^3^ A real‐world, retrospective study reported a mean treatment interval of 168.8 days between BoNT treatments in their pediatric spasticity cohort.^33^

Our study, which we believe is the largest on this topic to date, found issues with BoNT treatment persistency. Some spasticity studies have suggested factors such as adverse events, lack of efficacy, consent withdrawal, loss to follow‐up, travel distance required to receive treatment, lack of caregiver support, and alternative interventions may contribute to BoNT treatment discontinuation, but their findings were limited by small population samples.^26^, ^38^, ^39^, ^40^, ^41^, ^42^, ^43^, ^44^, ^45^ Other factors contributing to low BoNT persistency may include access and usage restrictions, such as prescription caps or reimbursement and insurance authorization difficulties.^46^ Improved communication in the therapeutic relationship between people living with spasticity and health care professionals could help to manage treatment expectations, goal setting,^47^ and potentially BoNT persistency. Findings from a prospective study showed that access to specialist spasticity services is often limited or nonexistent after a stroke; some patients may need to wait over 6 months, and most are discharged from secondary care without recommended therapy.^48^ Owing to the long‐term nature of PSS, there is a significant burden of this condition on patients, caregivers, and society.^49^ Ongoing studies, such as the EPITOME trial,^50^ may help improve understanding of the true PSS patient experience. [Correction added on 02 April 2026, after first online publication: In the previous sentence, “(Efficacy of Prednisone for the Treatment of Ocular Myasthenia)” has been deleted in this version.]

Based on the recommended retreatment regimen of 12 weeks for onaBoNT‐A in chronic migraine and eight to nine treatment cycles over the 2‐year period,^1^ the mean number of treatments was lower (4.7) and the time between treatments was longer (15 weeks) than expected in the migraine cohort, and only 19.7% of patients were considered persistent with treatment over the 2‐year period. In a retrospective chart review of patients with chronic migraine initiated on onaBoNT‐A over a 2‐year period, patients received treatment cycles >12 weeks apart on average, which the authors note likely reflects the real‐world situation of difficulty with scheduling and other factors that interfere with timely administration.^51^ The low persistency observed in this cohort was consistent with published real‐world studies in adults with migraine.^52^, ^53^ Reasons why patients with migraine may stop treatment include avoiding side effects, perceived lack of efficacy, availability of preventive medications, and lack of access to health care providers or insurance coverage.^54^, ^55^, ^56^, ^57^, ^58^

Of patients with bladder dysfunction, 17.6% were considered persistent with treatment over the 2‐year period using the time between treatment determined in this study (319 days), which may represent a typical retreatment schedule in these patients. Pivotal studies have shown that retreatment is expected about every 24 weeks in adults with overactive bladder and about every 42–48 weeks for adults with detrusor overactivity associated with a neurological condition.^1^ A review by Eldred‐Evans et al. stated that the majority of studies of idiopathic overactive bladder reported a BoNT‐A retreatment interval of 6.0–26.7 months. The authors note that retreatment interval may be confounded by multiple factors, including treatment delays and concomitant use of other medications, but also requests for retreatment by patients.^59^ In a study of patients with overactive bladder treated with onaBoNT‐A, the mean time to patient request for retreatment was 7.6 months,^32^ corresponding to one or two treatments in a 12‐month period.

Using a 120‐day look‐forward from treatment date, the observed peak at eight to nine treatment cycles in the cervical dystonia, adult spasticity, stroke, and migraine cohorts over the 2‐year period is in line with the expected BoNT treatment regimens in these indications (ie, once every 3–4 months). However, only 9.2%–24.7% of these patients received eight to nine treatment cycles, possibly highlighting the low number of persistent patients. This peak was not observed for the pediatric spasticity and bladder dysfunction cohorts, which is consistent with the discussed longer treatment intervals reported in these indications.

Despite the size of the present study, claims data do not provide insights into the potential reasons behind low BoNT persistency. Future studies should be designed to address this evidence gap, considering factors influencing physician, patient, and also caregiver decision‐making.

This study also provides insight into long‐term BoNT treatment; some patients received several BoNT treatment cycles during the study, suggesting continued benefit across repeat treatments. Given that BoNT treatment may be readministered based on the return of clinical symptoms,^1^, ^2^ increased intertreatment intervals compared with the usual 3–4 months may have been preferred by patients if their efficacy goals were met.^1^ As such, a 2‐year follow‐up period may not have been sufficient to observe persistency in all patients. The labels of the BoNT treatments indicated for the conditions studied state that retreatment should occur “if needed,”^1^, ^2^, ^3^, ^4^ suggesting that some patients will likely not be persistent.

As with all claims‐based studies, certain limitations should be acknowledged. The Merative MarketScan database does not capture clinical intent, treatment planning, or patient preferences, and therefore retreatment as early as 1 day after a previous injection was recorded as a new cycle. Although this scenario is unlikely in clinical practice, only a small proportion of patients were identified with such back‐to‐back treatment claims, and the large sample size together with the use of median values limits their influence on the overall results. Although this limitation should be considered when interpreting the precision of interval estimates, importantly, the expected peak in retreatment at 90–120 days was clearly observed and aligns with clinical expectations, supporting the validity and relevance of these findings to real‐world practice. Other limitations include that the quality of the Merative MarketScan data depends on the accuracy and completeness of the information system and is subject to potential bias, coding errors, and missing data. However, the eligibility criteria required that patients had a medical claim for BoNT, and specific diagnosis and treatment codes helped to mitigate potential errors. Only patients with continuous eligibility were included, and patients enrolled in other insurance plans during the study period were excluded. The findings may not be applicable to other commercially insured populations. Additionally, our analysis studied various disorders with different severity, progression, and treatment history, which complicated data interpretation.

Nevertheless, the study used a large, diverse, real‐world patient population, with longitudinal follow‐up, minimizing selection and reporting bias. Further studies are warranted to explore the reasons behind the low persistency with BoNT, the notable drop in the proportion of patients receiving BoNT treatment after the first injection cycle, and the longer interval between treatments in relation to the recommended treatment schedule.

## CONCLUSIONS

In this 2‐year follow‐up period retrospective analysis, a large proportion of patients receiving BoNT stopped treatment after the first injection cycle. Using a 120‐day look‐forward from treatment date, persistency with BoNT treatment over the 2‐year follow‐up period was low across all cohorts. Few patients in the adult spasticity and stroke cohorts received eight to nine treatments over 2 years (ie, once every 3–4 months, the recommended and labeled retreatment frequency for BoNT‐A in these conditions). Further research is needed to determine the underlying reasons for stopping treatment and to help to inform clinical strategies that can improve outcomes for patients receiving BoNT.

## FUNDING INFORMATION

This study was sponsored by Ipsen.

## DISCLOSURE

D.M.S. has received personal compensation for serving as a consultant and research grant support from Allergan/AbbVie, Ipsen, and Merz Pharma. J.B. and S.P. are employees and shareholders of Ipsen. S.G. has received personal compensation for serving as an employee of Ipsen and as a consultant for SSiPeople. A.T.P. has received personal compensation for serving as a consultant for Revance, on a scientific advisory or data safety monitoring board for Allergan and Ipsen, and on a Speaker's Bureau for Allergan/AbbVie and Ipsen. The institution of A.T.P. has received research support from Allergan, Ipsen, and Revance.

## INFORMED CONSENT STATEMENT

Given that this was a retrospective claims study in which all data were deidentified according to regulations, ethics approval and consent to participate were not required.

## Data Availability

Qualified researchers with a valid research question may request anonymized patient‐level data by contacting an Ipsen representative. Further information on Ipsen's Data Sharing policy is available here (https://www.ipsen.com/clinical-data-transparency/).

## Appendix

Botulinum toxin (or “BoNT”) is an injectable treatment used to help relax overactive muscles. Clinical guidelines recommend BoNT treatment to help improve health outcomes in various conditions affected by overactive muscles, such as spasticity and bladder dysfunction. Receiving BoNT treatment in the way that it is prescribed should help individuals get the maximum benefit of treatment.

Using anonymous national health insurance data from the USA (from the Merative® MarketScan database), we investigated how often patients received treatment over a 2‐year period i.e. how persistent they were with treatment. Patients were expected to receive BoNT treatment approximately every 3–4 months. We considered patients to be persistent with treatment if they did not have a gap longer than 120 days, approximately 4 months, between treatment cycles over the 2‐year period.

Our study included 19,711 individuals who received at least one BoNT treatment, had at least 2 years of follow‐up data available and had a diagnosis of adult spasticity (1,912), pediatric spasticity (638), stroke (611), cervical dystonia (688), migraine (7,778), or bladder dysfunction (1,791).

Based on the 120‐day period described above, we found that a substantial proportion of individuals across all diagnostic groups stopped BoNT treatment after only one treatment cycle. Overall, 31.1% of all patients received only one BoNT injection cycle in the 2‐year follow‐up. Few individuals received the expected 8–9 treatments over the 2‐year study period. The proportion was lowest for individuals with bladder dysfunction (0.3%) or pediatric spasticity (2.4%), and highest for those with migraine (24.7%) or cervical dystonia (15.4%). The average number of treatments received over the 2‐year study ranged from 2.1 in the bladder dysfunction group to 4.7 in the migraine group.

Further research is required to help understand why so many patients stop BoNT treatment early. Understanding these reasons could help to address the issues preventing them from benefiting fully from treatment. [Correction added on 02 April 2026, after first online publication: The section head “Appendix A” has been changed to “Plain Language Summary” in this version.]
